# Publisher Correction: Adaptive partitioning of a gene locus to the nuclear envelope in *Saccharomyces cerevisiae* is driven by polymer-polymer phase separation

**DOI:** 10.1038/s41467-023-38076-6

**Published:** 2023-04-21

**Authors:** Lidice González, Daniel Kolbin, Christian Trahan, Célia Jeronimo, François Robert, Marlene Oeffinger, Kerry Bloom, Stephen W. Michnick

**Affiliations:** 1 Département de Biochimie, Université de Montréal, C.P. 6128, Succursale centre-ville, Montréal, QC H3C 3J7 Canada; 2 Department of Biology, University of North Carolina at Chapel Hill, Chapel Hill, NC 27599 USA; 3 Institut de recherches cliniques de Montréal, 110 Avenue des Pins Ouest, Montréal, QC H2W 1R7 Canada; 4 Faculty of Medicine, Division of Experimental Medicine, McGill University, Montréal, QC H3A 1A3 Canada; 5 Département de Médecine, Faculté de Médecine, Université de Montréal, 2900 Boul. Édouard-Montpetit, Montréal, QC, H3T 1J4 Canada

**Keywords:** Chromatin remodelling, Gene expression, Gene regulation

Correction to: *Nature Communications* 10.1038/s41467-023-36391-6, published online 28 February 2023

The original version of this article contained formatting errors in the Peer Review file and the Supplementary Information. The Peer Review file and Supplementary Information file have been corrected.

## Supplementary information

Updated Peer Review File

Updated Supplementary Information

