# Supplementary material for: Adaptive partitioning of a gene locus to the nuclear envelope in Saccharomyces cerevisiae is driven by polymer-polymer phase separation
Source: Nat Commun. 2023 Feb 28;14:1135. doi: 10.1038/s41467-023-36391-6 (PMC9975218; doi:10.1038/s41467-023-36391-6)
Supplement: Supplementary file 1 — Supplementary Information [file 41467_2023_36391_MOESM1_ESM.pdf]

Supplementary information

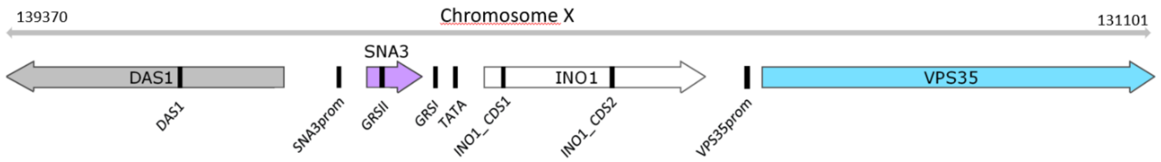

Supplementary Fig. 1

***INO1* and proximal loci positions in chromosome X**. Black bars indicate positions where the incorporation of newly synthesized FLAG-H3 into nucleosomes was measured by ChIP-qPCR assays.

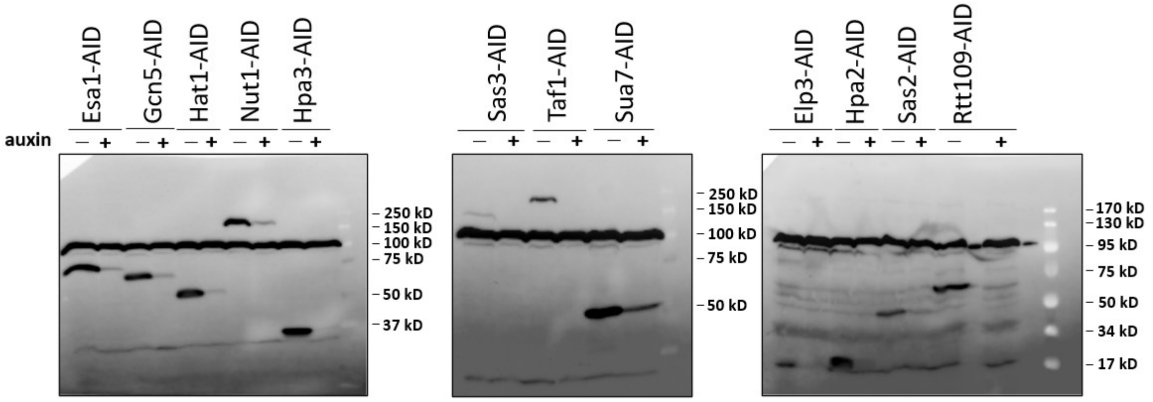

Supplementary Fig. 2

Detection of HATs-AID*-6FLAG proteins in the presence and absence of auxin. Protein levels of 12 different HATs carrying a C-terminal AID* tag extended by a FLAG epitope, analyzed by western blotting with an anti-Flag M2 antibody. Representative results of three independent experiments. Where indicated, cultures were treated with 500 µM auxin for 1 h 30 min before preparation of the lysates.

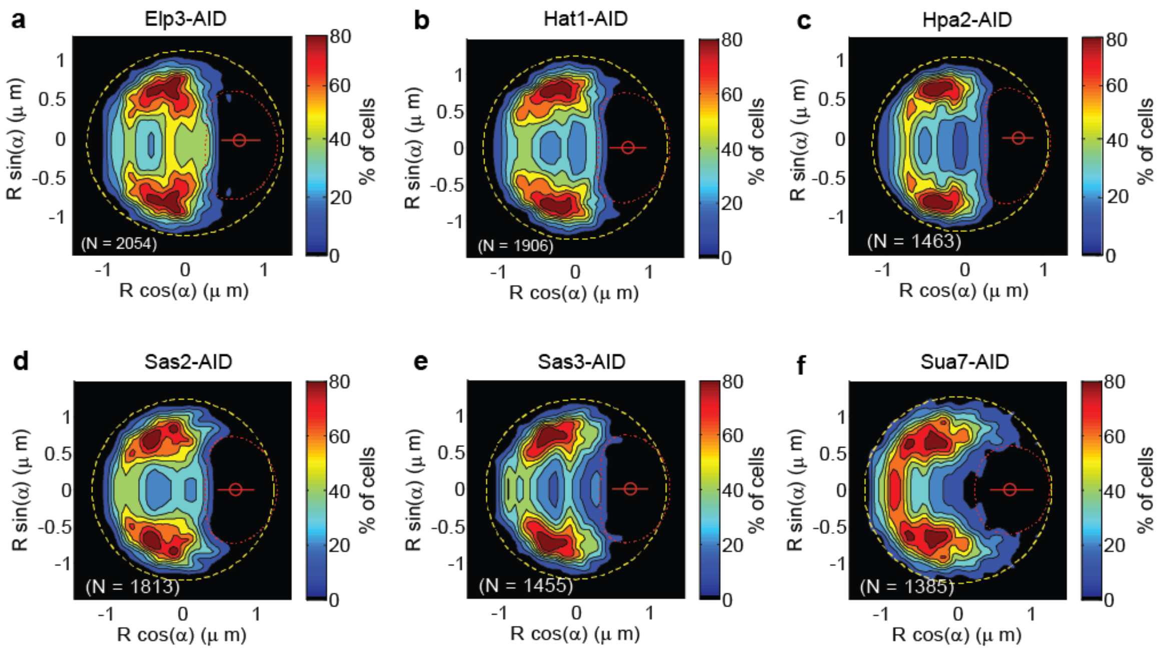

Supplementary Fig. 3

Statistical distribution of the *INO1* locus in strains expressing different HAT-AID grown in the absence of inositol (active) and after treating the cells with auxin. a-f *INO1* locus probability maps after nucloc software analysis of thousands of nuclei per strain.

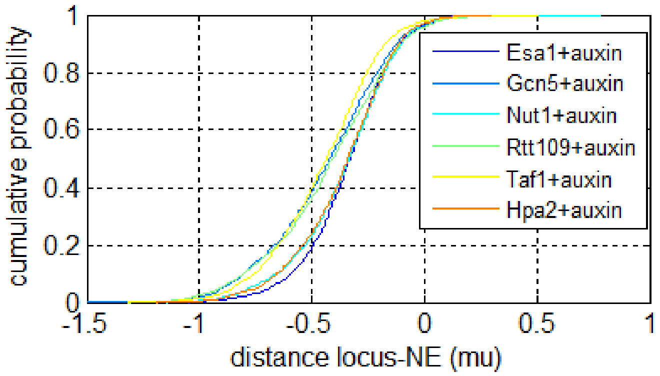

Supplementary Fig. 4

Cumulative distributions of distances to the nuclear envelope for the active *INO1* locus in six auxin-induced histone deacetylase degradation strains. Distributions shifted closer to 0 µm indicate that active loci are closer to the nuclear envelope. Source data are provided as a Source Data file.

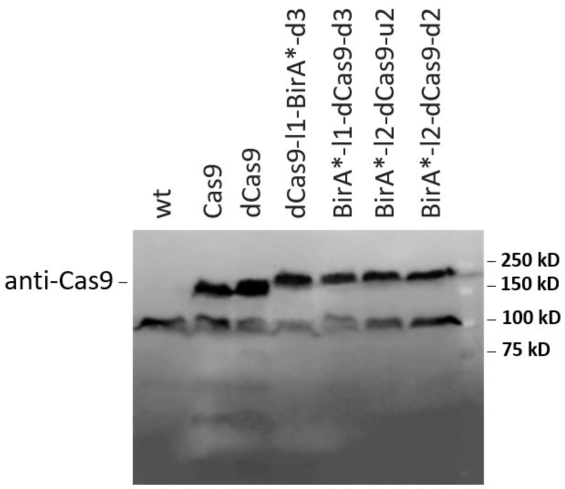

Supplementary Fig. 5

Detection of different Cas9 and BirA*-dCas9 fusions in yeast strains. Protein levels of Cas9, dCas9 and different BirA*-dCas9 fusions with BirA* in different N- or C-terminal positions and using short or long linker sequences, examined by western blotting with a mouse monoclonal Cas9 antibody 7A9-3A3 (Santa Cruz Biotechnology). Representative results of three independent experiments.

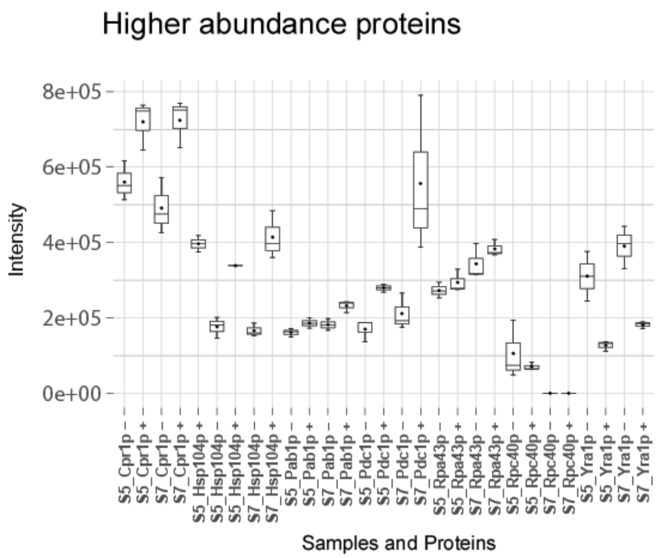
Higher abundance proteins
Intensity
Samples and Proteins

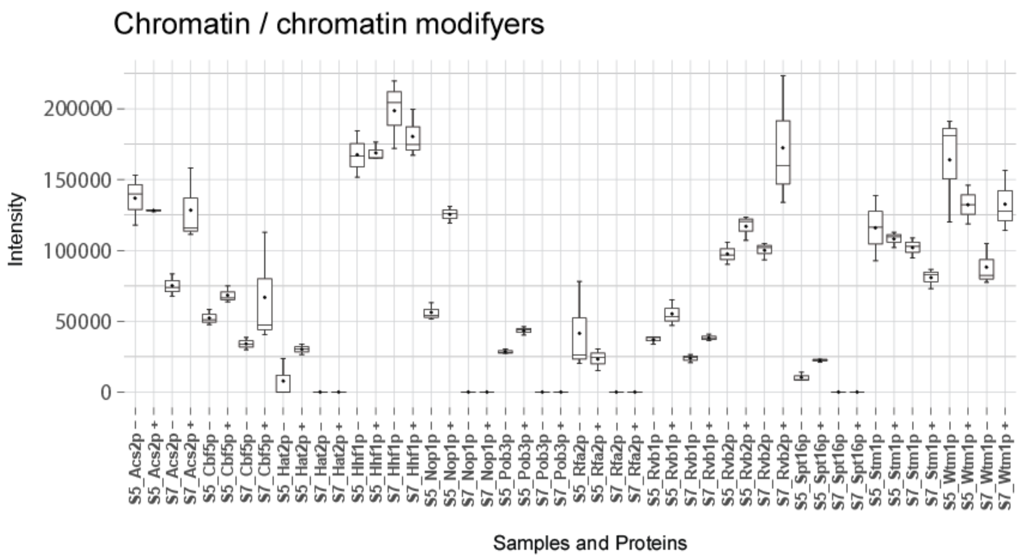
Chromatin / chromatin modifyers
Intensity
Samples and Proteins

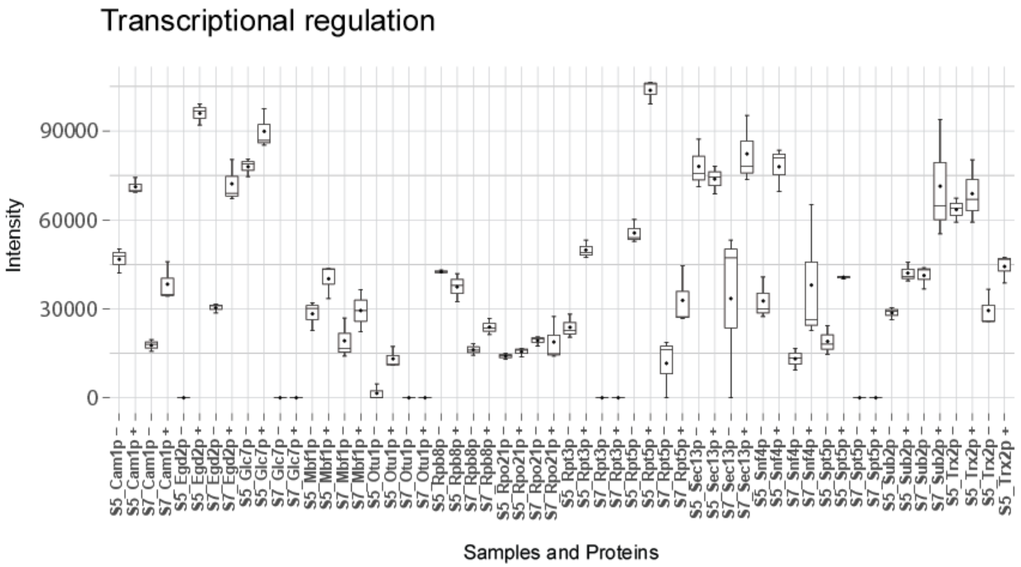
Transcriptional regulation
Intensity
Samples and Proteins

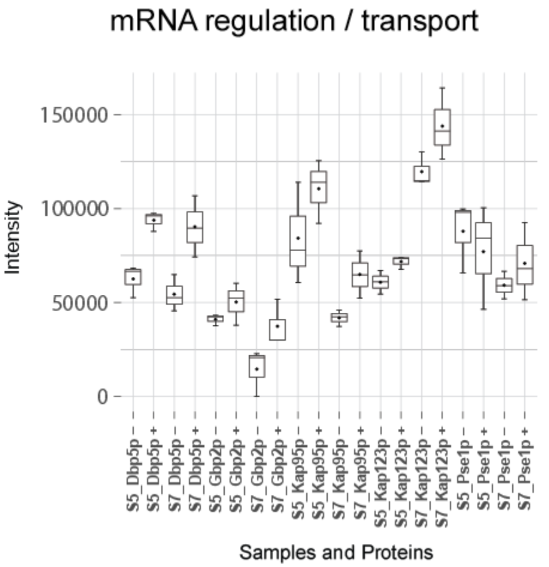
mRNA regulation / transport
Intensity
Samples and Proteins

Supplementary Fig. 6

Box plots of proteins intensities measured by MS for all proteins related to chromatin or transcription meeting quantification criteria. Purified protein measurements from biological triplicates expressing either gRNAs-BirA*-dCas9 (S5 and S7; +) along with their respective BirA*-dCas9 negative controls (S5 and S7; -) are shown side by side. Source data are provided as a Source Data file. Median is indicated as middle line, average as a black dot, 25th and 75th percentile as boxes and whiskers represent 5th and 95th percentile. For plotting, proteins were grouped by function, and proteins with significantly higher abundance compared to their corresponding group were plotted separately. See also Supplementary Data 4-6.

Supplementary Table 1 Yeast strains used in this study

Strain	Genotype	Reference
LMY52	*MAT***a** *ade2-1 can1-100 his3-11,15::LacI-GFP:HIS3 leu2-3,112 trp1-1 ura3-1 INO1:LacO128:URA3 SEC63-13myc::kan*^{r} *ADE2:Nup49GFP_Nop1RFP*	Brickner et al.[1]
JBY397	*MAT***a** *ade2-1 can1-100 his3-11,15::LacI-GFP:HIS3 leu2-3,112 trp1-1 ura3-1 INO1:LacO128:URA3 SEC63-13myc::kan*^{r}	Brickner et al.[2]
ML1	*MAT***a** *ade2-1 can1-100 his3-11,15::LacI-GFP:HIS3 leu2-3,112 trp1-1 ura3-1 INO1:LacO128:URA3 SEC63-13myc::kan*^{r} *ADE2:Nup49GFP_Nop1RFP grsIΔ* pWS171-sgRNA-GRSI	This study
ML2	*MAT***a** *ade2-1 can1-100 his3-11,15::LacI-GFP:HIS3 leu2-3,112 trp1-1 ura3-1 INO1:LacO128:URA3 SEC63-13myc::kan*^{r} *ADE2:Nup49GFP_Nop1RFP grsIIΔ* pWS171-sgRNA-GRSII	This study
ML3	*MAT***a** *ade2-1 can1-100 his3-11,15::LacI-GFP:HIS3 leu2-3,112 trp1-1 ura3-1 INO1:LacO128:URA3 SEC63-13myc::kan*^{r} *ADE2:Nup49GFP_Nop1RFP grsIΔ-grsIIΔ* pWS171-sgRNA-GRSI pWS175-sgRNA-GRSII	This study
YAN1001	*MAT***a** *ura3-52::HHF1*-p*GAL1/10*- Flag-*HHT1-URA3 lys2-801 his3Δ200 leu2Δ1* *hht1-hhf1*::*LEU2 hht2-hhf2*::HIS3 *trp1Δ63 bar1Δ::NATMX4* /pNOY 439 [CEN6 ARS4-TRP1 *HHF2* MYC-*HHT2*]	Rufiange et al.,[3]
ML4	*MAT***a** *ura3-52::HHF1*-p*GAL1/10*- Flag-*HHT1-URA3 lys2-801 his3Δ200 leu2Δ1* *hht1-hhf1*::*LEU2 hht2-hhf2*::HIS3 *trp1Δ63 bar1Δ::NATMX4* /pNOY 439 [CEN6 ARS4-TRP1 *HHF2* MYC-*HHT2*] *grsIΔ* pWS175-sgRNA-GRSI	This study
ML5	*MAT***a** *ura3-52::HHF1*-p*GAL1/10*- Flag-*HHT1-URA3 lys2-801 his3Δ200 leu2Δ1*	This study

	hht1-hhf1::*LEU2 hht2-hhf2*::HIS3 *trp1Δ63 bar1Δ::NATMX4* /pNOY 439 [CEN6 ARS4-TRP1 *HHF2* MYC-*HHT2*] *grsIIΔ* pWS175-sgRNA-GRSII	
ML6	*MAT***a** *ura3-52::HHF1*-p*GAL1/10-* Flag-*HHT1-URA3 lys2-801 his3Δ200 leu2Δ1* *hht1-hhf1*::*LEU2 hht2-hhf2*::HIS3 *trp1Δ63 bar1Δ::NATMX4* /pNOY 439 [CEN6 ARS4-TRP1 *HHF2* MYC-*HHT2*] *grsIΔ-grsIIΔ* pWS175-sgRNA-GRSI pWS175-sgRNA-GRSII	This study
ML7	*MAT***a** *ade2-1 can1-100 his3-11,15::LacI-GFP:HIS3 leu2-3,112 trp1-1 ura3-1 INO1:LacO128:URA3 SEC63-13myc::kan*^{r} *ADE2:Nup49GFP_Nop1RFP* pWS174	This study
ML8	*MAT***a** *ade2-1 can1-100 his3-11,15::LacI-GFP:HIS3 leu2-3,112 trp1-1 ura3-1 INO1:LacO128:URA3 SEC63-13myc::kan*^{r} *ADE2:Nup49GFP_Nop1RFP* pWS174-dCa9	This study
ML9	*MAT***a** *ade2-1 can1-100 his3-11,15::LacI-GFP:HIS3 leu2-3,112 trp1-1 ura3-1 INO1:LacO128:URA3 SEC63-13myc::kan*^{r} *ADE2:Nup49GFP_Nop1RFP* pWS174-dCas9-l1-BirA*	This study
ML10	*MAT***a** *ade2-1 can1-100 his3-11,15::LacI-GFP:HIS3 leu2-3,112 trp1-1 ura3-1 INO1:LacO128:URA3 SEC63-13myc::kan*^{r} *ADE2:Nup49GFP_Nop1RFP* pWS174-BirA*-l1-dCas9	This study
ML11	*MAT***a** *ade2-1 can1-100 his3-11,15::LacI-GFP:HIS3 leu2-3,112 trp1-1 ura3-1 INO1:LacO128:URA3 SEC63-13myc::kan*^{r} *ADE2:Nup49GFP_Nop1RFP* pWS174-BirA*-l2-dCas9	This study
ML12	*MAT***a** *ade2-1 can1-100 his3-11,15::LacI-GFP:HIS3 leu2-3,112 trp1-1 ura3-1 INO1:LacO128:URA3 SEC63-13myc::kan*^{r} *ADE2:Nup49GFP_Nop1RFP* pWS174-dCas9-l1-BirA*-d3	This study
ML13	*MAT***a** *ade2-1 can1-100 his3-11,15::LacI-GFP:HIS3 leu2-3,112 trp1-1 ura3-1 INO1:LacO128:URA3 SEC63-13myc::kan*^{r} *ADE2:Nup49GFP_Nop1RFP* pWS174-BirA*-l1-dCas9-d3	This study

ML14	*MAT***a** *ade2-1 can1-100 his3-11,15::LacI-GFP:HIS3 leu2-3,112 trp1-1 ura3-1 INO1:LacO128:URA3 SEC63-13myc::kan*^{r} *ADE2:Nup49GFP_Nop1RFP* pWS174-BirA*-l2-dCas9-u2	This study
ML15	*MAT***a** *ade2-1 can1-100 his3-11,15::LacI-GFP:HIS3 leu2-3,112 trp1-1 ura3-1 INO1:LacO128:URA3 SEC63-13myc::kan*^{r} *ADE2:Nup49GFP_Nop1RFP* pWS174-BirA*-l2-dCas9-d2	This study
ML16	*MAT***a** *ade2-1 can1-100 his3-11,15::LacI-GFP:HIS3 leu2-3,112 trp1-1::ADH1-AtTIR1*^{9myc}*:TRP1 ura3-1 INO1:LacO128:URA3 SEC63-13myc::kan*^{r} *ADE2:Nup49GFP_Nop1RFP*	This study
ML17	*MAT***a** *ade2-1 can1-100 his3-11,15::LacI-GFP:HIS3 leu2-3,112 trp1-1::ADH1-AtTIR1*^{9myc}*:TRP1 ura3-1 INO1:LacO128:URA3 SEC63-13myc::kan*^{r} *ADE2:Nup49GFP_Nop1RFP ELP3*$^{AID*\text{-}6FLAG::HygR}$	This study
ML18	*MAT***a** *ade2-1 can1-100 his3-11,15::LacI-GFP:HIS3 leu2-3,112 trp1-1::ADH1-AtTIR1*^{9myc}*:TRP1 ura3-1 INO1:LacO128:URA3 SEC63-13myc::kan*^{r} *ADE2:Nup49GFP_Nop1RFP ESA1*$^{AID*\text{-}6FLAG::HygR}$	This study
ML19	*MAT***a** *ade2-1 can1-100 his3-11,15::LacI-GFP:HIS3 leu2-3,112 trp1-1::ADH1-AtTIR1*^{9myc}*:TRP1 ura3-1 INO1:LacO128:URA3 SEC63-13myc::kan*^{r} *ADE2:Nup49GFP_Nop1RFP GCN5*$^{AID*\text{-}6FLAG::HygR}$	This study
ML20	*MAT***a** *ade2-1 can1-100 his3-11,15::LacI-GFP:HIS3 leu2-3,112 trp1-1::ADH1-AtTIR1*^{9myc}*:TRP1 ura3-1 INO1:LacO128:URA3 SEC63-13myc::kan*^{r} *ADE2:Nup49GFP_Nop1RFP HAT1*$^{AID*\text{-}6FLAG::HygR}$	This study
ML21	*MAT***a** *ade2-1 can1-100 his3-11,15::LacI-GFP:HIS3 leu2-3,112 trp1-1::ADH1-AtTIR1*^{9myc}*:TRP1 ura3-1 INO1:LacO128:URA3 SEC63-13myc::kan*^{r} *ADE2:Nup49GFP_Nop1RFP HPA2*$^{AID*\text{-}6FLAG::HygR}$	This study
ML22	*MAT***a** *ade2-1 can1-100 his3-11,15::LacI-GFP:HIS3 leu2-3,112 trp1-1::ADH1-AtTIR1*^{9myc}*:TRP1 ura3-1 INO1:LacO128:URA3 SEC63-13myc::kan*^{r} *ADE2:Nup49GFP_Nop1RFP HPA3*$^{AID*\text{-}6FLAG::HygR}$	This study
ML23	*MAT***a** *ade2-1 can1-100 his3-11,15::LacI-GFP:HIS3 leu2-3,112 trp1-1::ADH1-AtTIR1*^{9myc}*:TRP1 ura3-1 INO1:LacO128:URA3 SEC63-13myc::kan*^{r} *ADE2:Nup49GFP_Nop1RFP NUT1*$^{AID*\text{-}6FLAG::HygR}$	This study

ML24	*MAT***a** *ade2-1 can1-100 his3-11,15::LacI-GFP:HIS3 leu2-3,112 trp1-1::ADH1-AtTIR1*^{9myc}*:TRP1 ura3-1 INO1:LacO128:URA3 SEC63-13myc::kan*^{r} *ADE2:Nup49GFP_Nop1RFP RTT109*$^{AID*\text{-}6FLAG::HygR}$	This study
ML25	*MAT***a** *ade2-1 can1-100 his3-11,15::LacI-GFP:HIS3 leu2-3,112 trp1-1::ADH1-AtTIR1*^{9myc}*:TRP1 ura3-1 INO1:LacO128:URA3 SEC63-13myc::kan*^{r} *ADE2:Nup49GFP_Nop1RFP SAS2*$^{AID*\text{-}6FLAG::HygR}$	This study
ML26	*MAT***a** *ade2-1 can1-100 his3-11,15::LacI-GFP:HIS3 leu2-3,112 trp1-1::ADH1-AtTIR1*^{9myc}*:TRP1 ura3-1 INO1:LacO128:URA3 SEC63-13myc::kan*^{r} *ADE2:Nup49GFP_Nop1RFP SAS3*$^{AID*\text{-}6FLAG::HygR}$	This study
ML27	*MAT***a** *ade2-1 can1-100 his3-11,15::LacI-GFP:HIS3 leu2-3,112 trp1-1::ADH1-AtTIR1*^{9myc}*:TRP1 ura3-1 INO1:LacO128:URA3 SEC63-13myc::kan*^{r} *ADE2:Nup49GFP_Nop1RFP SUA7*$^{AID*\text{-}6FLAG::HygR}$	This study
ML28	*MAT***a** *ade2-1 can1-100 his3-11,15::LacI-GFP:HIS3 leu2-3,112 trp1-1::ADH1-AtTIR1*^{9myc}*:TRP1 ura3-1 INO1:LacO128:URA3 SEC63-13myc::kan*^{r} *ADE2:Nup49GFP_Nop1RFP TAF1*$^{AID*\text{-}6FLAG::HygR}$	This study

Supplementary Table 2 Plasmids used in this study

Plasmid	Description	Reference
pWS082	sgRNA entry vector	Addgene 90516, Shaw *et al.* [4]
pWS082-GRSI	sgRNA GRSI	This study
pWS082-GRSII	sgRNA GRSII	This study
pWS171	Cas9 gap repair vector - LEU2	Addgene 90518, Shaw *et al.* [4]
pWS175	Cas9 gap repair vector - Hyg^R	Addgene 90962, Shaw *et al.* [4]
pWS174	Cas9 gap repair vector - Nat^R	Addgene 90961, Shaw *et al.* [4]
pME4478	ASC1-birA*	Opitz *et al.,* [5]
pWS082-GRSI-u2	sgRNA GRSI upstream 2	This study
pWS082-GRSI-d2	sgRNA GRSI downstream 2	This study
pWS082-GRSI-d3	sgRNA GRSI downstream 3	This study
pWS174-dCas9	dCas9 vector – Nat^R	This study
pWS174-dCas9-l1-BirA*	dCas9 vector – Nat^R C-terminal fused by linker 1 to BirA*	This study
pWS174-BirA*-l1-dCas9	dCas9 vector – Nat^R N-terminal fused by linker 1 to BirA*	This study
pWS174-BirA*-l2-dCas9	dCas9 vector – Nat^R N-terminal fused by linker 2 to BirA*	This study
YIp204-PADH1-atTIR1-9myc	F-box protein TIR1-9myc (for use with the AID degron system)	Addgene 99532, unpublished Ulrich lab
pHyg–AID*–6FLAG	C-terminal AID*-6FLAG degron cassette - Hyg^R	Morawska and Ulrich [6]

Supplementary Table 3 Samples used for mass spectrometry analysis

Sample	Name	Description	Controls correspondence
S2	BirA*-l1-dCas9	BirA* N-terminal to dCas9, short linker	Negative for sample S7
S3	BirA*-l2-dCas9	BirA* N-terminal to dCas9, long linker	Negative for samples S5, S6
S4	dCas9-l1-BirA*	BirA* C-terminal to dCas9, short linker	Negative for sample S8
S5	BirA*-l2-dCas9-u2	BirA* N-terminal to dCas9, long linker, guide u2 (24bp upstream GRSI site)	
S6	BirA*-l2-dCas9-d2	BirA* N-terminal to dCas9, long linker, guide d2 (35bp downstream GRSI site)	
S7	BirA*-l1-dCas9-d3	BirA* N-terminal to dCas9, short linker, guide d3 (15bp downstream GRSI site)	
S8	dCas9-l1-BirA*-d3	BirA* C-terminal to dCas9, short linker, guide d3 (15bp downstream GRSI site)	

Supplementary Table 4 Primer sequences used for ChIP-qPCR and RT-qPCR experiments

Name	Sequence	Experiment	Reference
DAS1 CDS_Fw	TTCTCGGCTTTCTCATATGACTT	ChIP-qPCR and RT-qPCR	This study
DAS1 CDS_Rev	ACTCCCTCCTCCAATGCAATTT	ChIP-qPCR and RT-qPCR	This study
SNA3prom_Fw	CGGGGGAAAAGGTCTCACTA	ChIP-qPCR	This study
SNA3prom_Rev	GTTGTTTGCTTTCTGCTGGG	ChIP-qPCR	This study
GRSIIqPCR_Fw	AGCGTAAGGGTATGTTCAACAGG	ChIP-qPCR and RT-qPCR	This study
GRSIIqPCR_Rev	ACGAACGTTCACTACTCGTTTCA	ChIP-qPCR and RT-qPCR	This study
GRSIqPCR_Fw	ATGAAATACGTGCCGGTGTTC	ChIP-qPCR	This study
GRSIqPCR_Rev	ACTTTTCACATGCCGCATTTAGC	ChIP-qPCR	This study
INO1TATA_Fw	TCACATGGAGCAGAGAAAGC	ChIP-qPCR	This study
INO1TATA_Rev	GGAACCCGACAACAGAACAA	ChIP-qPCR	This study
INO1CDS1_Fw	GACAAGTGCACGTACAAGGA	ChIP-qPCR and RT-qPCR	Similar to INO1CDS F in Brickner *et al.,* 2007[7]
INO1CDS1_Rev	TCTTGAACAGTGGGCGTTAC	ChIP-qPCR and RT-qPCR	Similar to INO1CDS R Brickner *et al.,* 2007[7]
INO1CDS2_Fw	GCTCCTTCCACGATCTTTGC	ChIP-qPCR	This study
INO1CDS2_Rev	GTACCCTCATGCTCAGCCAG	ChIP-qPCR	This study
VPS35prom_Fw	AGGTAGATGCGAGAAAGTGCT	ChIP-qPCR	This study
VPS35prom_Rev	TCACTGGGCCTTGTTGAGTG	ChIP-qPCR	This study
VPS35CDS_Fw	GGAGGACGATCCGTCGTTTA	RT-qPCR	This study
VPS35CDS_Rev	TTTCCCTCTCACGAAGTGGC	RT-qPCR	This study
ACT1CDS F	GGTTATTGATAACGGTTCTGGTATG	RT-qPCR	Brickner *et al.,* 2007[7]
ACT1CDS R	ATGATACCTTGGTGTCTTGGTCTAC	RT-qPCR	Brickner *et al.,* 2007[7]

References

1 Brickner, D. G. *et al.* Transcription factor binding to a DNA zip code controls interchromosomal clustering at the nuclear periphery. *Developmental cell* **22**, 1234-1246, doi:10.1016/j.devcel.2012.03.012 (2012).

2 Brickner, J. H. & Walter, P. Gene recruitment of the activated INO1 locus to the nuclear membrane. *PLoS biology* **2**, e342, doi:10.1371/journal.pbio.0020342 (2004).

3 Rufiange, A., Jacques, P. E., Bhat, W., Robert, F. & Nourani, A. Genome-wide replication-independent histone H3 exchange occurs predominantly at promoters and implicates H3 K56 acetylation and Asf1. *Mol Cell* **27**, 393-405, doi:10.1016/j.molcel.2007.07.011 (2007).

4 Shaw, W. M. *et al.* Engineering a Model Cell for Rational Tuning of GPCR Signaling. *Cell* **177**, 782-796.e727, doi:https://doi.org/10.1016/j.cell.2019.02.023 (2019).

5 Opitz, N. *et al.* Capturing the Asc1p/Receptor for Activated C Kinase 1 (RACK1) Microenvironment at the Head Region of the 40S Ribosome with Quantitative BioID in Yeast. *Molecular & cellular proteomics : MCP* **16**, 2199-2218, doi:10.1074/mcp.M116.066654 (2017).

6 Morawska, M. & Ulrich, H. D. An expanded tool kit for the auxin-inducible degron system in budding yeast. *Yeast (Chichester, England)* **30**, 341-351, doi:10.1002/yea.2967 (2013).

7 Brickner, D. G. *et al.* H2A.Z-mediated localization of genes at the nuclear periphery confers epigenetic memory of previous transcriptional state. *PLoS biology* **5**, e81, doi:10.1371/journal.pbio.0050081 (2007).
