# Supplementary material for: Adaptive partitioning of a gene locus to the nuclear envelope in Saccharomyces cerevisiae is driven by polymer-polymer phase separation
Source: Nat Commun. 2023 Feb 28;14:1135. doi: 10.1038/s41467-023-36391-6 (PMC9975218; doi:10.1038/s41467-023-36391-6)
Supplement: Supplementary file 3 — Description of Additional Supplementary Files [file 41467_2023_36391_MOESM3_ESM.pdf]

Description of Additional Supplementary Files

Note: Supplementary Data 1-6 provide in a ZIP file.

File name: Supplementary Data 1 (separate file)

Description: Raw Spectronaut output of the mass spectrometry analysis between biological triplicates of BirA*-l2-dCas9-u2 (samples S5) and corresponding negative controls BirA*-l2-dCas9 (samples S3). A two-sided unpaired Student's t-test with equal variance was used to determine significant protein enrichment from sgRNA-BirA*-dCas9 triplicates.

File name: Supplementary Data 2 (separate file)

Description: Raw Spectronaut output of the mass spectrometry analysis between biological triplicates of BirA*-l1-dCas9-d3 (samples S7) and corresponding negative controls BirA*-l1-dCas9 (samples S2). A two-sided unpaired Student's t-test with equal variance was used to determine significant protein enrichment from sgRNA-BirA*-dCas9 triplicates.

File name: Supplementary Data 3 (separate file)

Description: Protein candidates meeting quantification requirements as described in the mass spectrometry method section for samples 5 and 3 biological triplicates, along with their intensities, average intensities, standard deviations, variation coefficients, fold changes, and associated *p*-values. A two-sided unpaired Student's t-test with equal variance was used to determine significant protein enrichment from sgRNA-BirA*-dCas9 triplicates.

File name: Supplementary Data 4 (separate file)

Description: Protein candidates meeting quantification requirements as described in the mass spectrometry method section for samples 7 and 2 biological triplicates, along with their intensities, average intensities, standard deviations, variation coefficients, fold changes, and associated *p*-values. A two-sided unpaired Student's t-test with equal variance was used to determine significant protein enrichment from sgRNA-BirA*-dCas9 triplicates.

File name: Supplementary Data 5 (separate file)

Description: Protein candidates list from both mass spectrometry datasets matching the Uniprot chromatin and transcription related proteins list, along with their ontologies.

File name: Supplementary Data 6 (separate file)

Description: Uniprot search output of chromatin and transcription related proteins in *S. cerevisiae*, along with their ontologies.

File name: Supplementary Movie 1

Description: ChromoShake simulation of 2µm heterogeneous DNA chain.

Related to Fig. 5. This video shows a single run of ChromoShake simulation for 2um of DNA modeled as a bead-springs polymer with regions of heterogenous persistence length. Simulation begins with an extended linear polymer chain that quickly collapses to an equilibrium conformation due to thermal fluctuations and inherent (modelled) physical parameters. The segments of persistence length 250 nm are flanking the middle segment, which has persistence length of 5 nm and contains a highlighted region (green beads) to which all the statistical analyses in this paper (MSDs, spring constants, radii of

gyration, and aspect ratio) are applied. Positions of blue beads are used for reference only and are not included in the statistical calculation.
