# Supplementary material for: Adaptive partitioning of a gene locus to the nuclear envelope in Saccharomyces cerevisiae is driven by polymer-polymer phase separation
Source: Nat Commun. 2023 Feb 28;14:1135. doi: 10.1038/s41467-023-36391-6 (PMC9975218; doi:10.1038/s41467-023-36391-6)
Supplement: Supplementary file 6 — Reporting Summary [file 41467_2023_36391_MOESM6_ESM.pdf]

nature portfolio

Corresponding author(s): Stephen W Michnick

Last updated by author(s): Dec 29, 2022

Reporting Summary

Nature Portfolio wishes to improve the reproducibility of the work that we publish. This form provides structure for consistency and transparency in reporting. For further information on Nature Portfolio policies, see our Editorial Policies and the Editorial Policy Checklist.

Statistics

For all statistical analyses, confirm that the following items are present in the figure legend, table legend, main text, or Methods section.

n/a	Confirmed	
☐	☒	The exact sample size (*n*) for each experimental group/condition, given as a discrete number and unit of measurement
☐	☒	A statement on whether measurements were taken from distinct samples or whether the same sample was measured repeatedly
☐	☒	The statistical test(s) used AND whether they are one- or two-sided<br>*Only common tests should be described solely by name; describe more complex techniques in the Methods section.*
☒	☐	A description of all covariates tested
☒	☐	A description of any assumptions or corrections, such as tests of normality and adjustment for multiple comparisons
☐	☒	A full description of the statistical parameters including central tendency (e.g. means) or other basic estimates (e.g. regression coefficient) AND variation (e.g. standard deviation) or associated estimates of uncertainty (e.g. confidence intervals)
☐	☒	For null hypothesis testing, the test statistic (e.g. *F*, *t*, *r*) with confidence intervals, effect sizes, degrees of freedom and *P* value noted<br>*Give P values as exact values whenever suitable.*
☒	☐	For Bayesian analysis, information on the choice of priors and Markov chain Monte Carlo settings
☒	☐	For hierarchical and complex designs, identification of the appropriate level for tests and full reporting of outcomes
☒	☐	Estimates of effect sizes (e.g. Cohen's *d*, Pearson's *r*), indicating how they were calculated

Our web collection on statistics for biologists contains articles on many of the points above.

Software and code

Policy information about availability of computer code

Data collection	Zen Blue edition (version 2.6), Zen Black edition, QuantStudio™ Real-Time PCR Software (version 1.2), LightCycler 96 software (version 1.1).
Data analysis	Nucloc software (version 01.06), WaveTracer tool in MetaMorph software (version 7.10.2.240), Fiji software (Image J, version 2.9.0), ChromoShake software (version 1.2.0), MATLAB (version R2021), LightCycler 96 software (version 1.1), Microsoft Excel, Spectronaut (version 15.1.210713), RStudio (version 2022.07.2), GraphPad Prism 9 version 9.5.0.<br>All original code for matlab methods specifically referenced in the text are accessible in a GitHub repository publicly available: https://github.com/kolbincode/INO1-paper. DOI: 10.5281/zenodo.7492891.

For manuscripts utilizing custom algorithms or software that are central to the research but not yet described in published literature, software must be made available to editors and reviewers. We strongly encourage code deposition in a community repository (e.g. GitHub). See the Nature Portfolio guidelines for submitting code & software for further information.

Data

Policy information about availability of data

All manuscripts must include a data availability statement. This statement should provide the following information, where applicable:

- Accession codes, unique identifiers, or web links for publicly available datasets
- A description of any restrictions on data availability
- For clinical datasets or third party data, please ensure that the statement adheres to our policy

Data availability statement:
Mass spectrometric data are publicly available via ProteomeXchange. Project accession: PXD029913. Project DOI: Not applicable. Reviewer account details: Username: reviewer_pxd029913@ebi.ac.uk<mailto:reviewer_pxd029913@ebi.ac.uk>.
The data supporting the results presented in this work are available in the main article and the supplementary information. Any additional information is available upon request from the corresponding author.

Human research participants

Policy information about studies involving human research participants and Sex and Gender in Research.

Reporting on sex and gender	NA
Population characteristics	NA
Recruitment	NA
Ethics oversight	NA

Note that full information on the approval of the study protocol must also be provided in the manuscript.

Field-specific reporting

Please select the one below that is the best fit for your research. If you are not sure, read the appropriate sections before making your selection.

☒ Life sciences ☐ Behavioural & social sciences ☐ Ecological, evolutionary & environmental sciences

For a reference copy of the document with all sections, see nature.com/documents/nr-reporting-summary-flat.pdf

Life sciences study design

All studies must disclose on these points even when the disclosure is negative.

Sample size	Sample size was selected based on standards for similar experiments performed before, considering it would be sufficient to perform statistical analysis. For statistical maps of the locus localization, as recommended by the designers of nucloc software thousands of nuclei per condition per strain were analyzed. For tracking and calculation of physical properties for the fluorescent locus, around 50 cells per condition per strain were processed. For the rest of the experiments at least 3 independent biological samples per condition were used.
Data exclusions	No data was excluded from the analyses.
Replication	Experiments were performed with at least 3 biological replicates. Attempts to replicate experimental results were successful.
Randomization	Samples were allocated randomly for treatments and analyses within the corresponding strain group.
Blinding	NA

Reporting for specific materials, systems and methods

We require information from authors about some types of materials, experimental systems and methods used in many studies. Here, indicate whether each material, system or method listed is relevant to your study. If you are not sure if a list item applies to your research, read the appropriate section before selecting a response.

Materials & experimental systems

n/a	Involved in the study
☐	☒ Antibodies
☒	☐ Eukaryotic cell lines
☒	☐ Palaeontology and archaeology
☒	☐ Animals and other organisms
☒	☐ Clinical data
☒	☐ Dual use research of concern

Methods

n/a	Involved in the study
☒	☐ ChIP-seq
☒	☐ Flow cytometry
☒	☐ MRI-based neuroimaging

Antibodies

Antibodies used

Mouse Monoclonal anti-FLAG M2 antibody (F1804, Sigma), lot# SLBT6752
Rabbit Polyclonal Anti-acetyl-Histone H3 (Lys14) Antibody (07-353, Millipore), Lot# 3776328
Rabbit Polyclonal Anti-Histone H3 antibody (ab1791, Abcam), Lot# GR3427866-1
Mouse Monoclonal Anti-Cas9 Antibody (7A9-3A3) (sc-517386, Santa Cruz Biotechnology), lot# G2519

Validation

anti-FLAG M2 antibody (F1804, Sigma). Application: validated in WB, IP, IHC, IF, ChIP, ChIP-seq. Reactivity: human, Drosophila, yeast (S. cerevisiae), bacteria. References: ChIP-seq, WB PMC9726891, ChIP, PMC9249852.
Anti-acetyl-Histone H3 (Lys14) Antibody (07-353, Millipore). Application: validated in ChIP, WB, ChIP-seq, DB. Reactivity: Human, Yeast (S. cerevisiae). References: ChIP PMID: 31365865, ChIP PMC4302307.
Rabbit Polyclonal Anti-Histone H3 antibody (ab1791, Abcam). Application: validated in ChIP, IP, WB, IF, IHC-P. Reactivity: Mouse, Rat, Human, Yeast (S. cerevisiae). References: ChIP PMID: 31365865, WB PMC7840730.
Mouse Monoclonal Anti-Cas9 Antibody (7A9-3A3) (sc-517386, Santa Cruz Biotechnology). Application: validated in WB, IP, IF, IHC-P, FCM. Reactivity: human, mice, yeast, bacteria. References: WB PMC9278866, WB PMC9005855.
